# Influencing Mechanisms of Prior Cold Deformation on Mixed Grain Boundary Network in the Thermal Deformation of Ni80A Superalloy

**DOI:** 10.3390/ma15186426

**Published:** 2022-09-16

**Authors:** Yu-Qing Zhang, Guo-Zheng Quan, Jiang Zhao, Wei Xiong

**Affiliations:** 1 Chongqing Key Laboratory of Advanced Mold Intelligent Manufacturing, School of Material Science and Engineering, Chongqing University, Chongqing 400044, China; 2 State Key Laboratory of Materials Processing and Die & Mould Technology, Huazhong University of Science and Technology, Wuhan 430074, China; 3 Key Laboratory of Advanced Reactor Engineering and Safety of Ministry of Education, Collaborative Innovation Center of Advanced Nuclear Energy Technology, Institute of Nuclear and New Energy Technology, Tsinghua University, Beijing 100084, China

**Keywords:** Ni80A superalloy, dynamic recrystallization, microstructure evolution, prior cold deformation, twin density, grain size

## Abstract

Within the grain boundary engineering (GBE) of alloys, a mixed grain boundary network with random grain boundaries interrupted by twin boundaries, contributes to enhancing the overall grain boundary-related properties. The higher density of twin boundaries is pursued herein. Furthermore, a two-stage deformation method, i.e., prior cold deformation followed by thermal deformation, was proposed for improving the mixed grain boundary network in the thermal deformation of Ni80A superalloy. The influence of prior cold deformation on the mixed grain boundary network was investigated through a series of two-stage deformation experiments. The analysis of the stress–strain curves shows that the critical strain for dynamic recrystallization (DRX) and peak strains decrease significantly under the effect of prior cold deformation. In comparison to the necklace-like microstructures that occur after a single thermal deformation, the microstructures apparent after a two-stage deformation are characterized by finer DRX grains with abundant Σ3^n^ twin boundaries, with a significantly improved density of the Σ3^n^ twin boundaries (BLD_Σ3_^n^) by a factor of around nine. With increasing prior cold strain, the grain size, after a two-stage deformation, decreases continuously, while the BLD_Σ3_^n^ increases firstly and then decreases. The mechanisms for improving the mixed grain boundary network via two-stage deformation were uncovered. The sub-grain boundaries formed in prior cold deformation stimulate the nucleation of DRX grains and twins; meanwhile, the driving force for grain boundary migration is enhanced due to prior stored energy. Then, DRX is activated in advance and occurs more completely, thereby promoting the formation of Σ3^n^ twin boundaries.

## 1. Introduction

Nickel-based alloys, especially the Ni80A superalloy, have been extensively deployed in the hot-section components of marine diesel engines, which are usually manufactured by thermal forming processes such as hot-rolling, forging, electric-upsetting, etc. [1,2,3]. Due to the long-term thermo-mechanical load and severe corrosion environs, the superior elevated-temperature performance of alloys, such as high-strength, strong corrosion-resistance, and excellent creep-resistance, are required. Grain boundary engineering (GBE), introduced in the 1980s by Watanabe [4], is considered a particularly attractive method to enhance the desirable properties of alloys. It has been successfully applied in many alloys with a low/medium stacking fault energy (SFE) [5,6,7]. The general principle of GBE is to modify the grain boundary character distribution (GBCD) and promote the formation of special grain boundaries in the microstructures of the alloys. The special grain boundaries, Σ3^n^ twin boundaries (*n* = 1, 2, 3), have relatively coherent interfaces that contain comparatively fewer vacancies and defects, resulting in low energy, the so-called “low-energy twin boundaries” [5,7,8]. It has been proven that these Σ3^n^ twin boundaries can modify the interconnectivity of a random high-angle grain boundary network [9,10], forming a mixed grain boundary network. Additionally, the sufficiently high density of Σ3^n^ twin boundaries contributes to enhancing grain boundary-related properties, such as the intergranular cracking-resistance [11], corrosion-resistance [12] and creep-resistance [13] of the alloys. Therefore, achieving a mixed grain boundary network with higher twin densities in the thermal deformation process is an effective approach to strengthening the comprehensive properties of these alloys.

During the thermal deformation process of an alloy, dynamic recrystallization (DRX), grain growth, and twin evolution mechanisms coexist and interact with each other. The evolution of twin density is governed by the comprehensive effects of these mechanisms. In order to improve the mixed grain boundary network, it is a prerequisite to describe the microstructure-evolution mechanisms associated with Σ3^n^ twin boundaries. Shi et al. [14], Aghaie-Khafri et al. [15], Ebrahimi et al. [16], Li et al. [17], etc., investigated the hot deformation behaviors and microstructure evolution of GH690, Inconel718, Monel400 Ni-Cu, and 625 alloys through isothermal compression tests. The results show that DRX is activated by the formation of twin boundaries, and the thermal deformation parameters, including strain, temperature, and strain rate, have a significant influence on the nucleation and growth of DRX grains and the evolution of twin boundaries. Jiang et al. [18] investigated the evolution of sub-structures and twin boundaries in the hot deformation process of 617B alloys. The microstructure characterization results confirmed that the formation of twins can promote the occurrence of the DRX process by accelerating the bulging and separation of the original grain boundaries. In addition, in order to predict twin density evolution in the dynamic hot deformation process, some researchers have focused on developing a mathematical model of twin density related to the processing or microstructural parameters of nickel-based alloys. Shi et al. [19] constructed an inversely proportional relationship between twin density and DRX grain size during the DRX process of Ni80A superalloy. This indicates that the probability of forming twins during grain boundary migration increases with the occurrence of DRX, while it decreases with the growth of the recrystallized grains. Mandal et al. [20] found that the density of Σ3 twin boundaries increases firstly and then decreases with an increasing DRX volume fraction and the recrystallized grain size during the hot compression process of 617 alloys. Martin Detrois et al. [21] and Quan et el. [22] developed the improved twin density models by introducing stored energy to quantitatively describe the evolution of twin boundary density in the thermal deformation processes of RR1000 and Ni80A superalloys, respectively. The results suggest that the mixed grain boundary network with finer grains and higher twin density usually corresponds to higher stored energy and the DRX fraction.

However, it is noteworthy that the DRX cannot occur fully in all cases where a specimen has deformed under a wide deformation parameter range. At a relatively low temperature and a mediate strain rate of 0.1 s^−1^, the grain boundary migration rate is relatively slow, and there is not enough stored energy to sufficiently activate the DRX process, resulting in a lower nucleation rate of the DRX grains and twins [14,15,20,22,23,24,25,26]. Thus, only the necklace-like microstructures with lower twin densities are obtained. At a higher temperature, although DRX occurs more completely under the relatively high thermal activation energy, stronger grain boundary mobility makes the recrystallized grains grow more [20,27,28,29]. This is not favorable for the nucleation of twins, leading to the mixed grain boundary network with coarse grains and lower twin density. Hence, it is significant to explore a new method to achieve the mixed grain boundary network with higher twin density in the thermal deformation of nickel-based alloys.

The primary processing route for GBE is a thermo-mechanical process (TMP), i.e., a single step or multiple iterative cold deformations followed by short-time annealing treatments [6,30,31]. This process usually can be divided into two categories, including strain-annealing and strain-recrystallization, and both of these can increase the proportion of Σ3^n^ twin boundaries. Until now, most investigators have analyzed the effects of the TMP processing parameters on twin boundary evolution, such as pre-deformation amount, annealing temperature, annealing time, iterative times, etc. Wang et al. [32] processed a nickel-based alloy via cold-rolling using different strains followed by annealing and reported that the stored energy in the cold-rolling and grain size plays a determinant role in the formation of annealing twins. Zhang et al. [33] investigated the evolution of grain boundary network and grain-size distribution using different prior deformation levels and annealing temperatures for nickel-based alloys. The results show that the density of the Σ3 twin boundary increases with an increasing prior deformation level and annealing temperature. Xia et al. [34] discussed the effects of cold-rolling deformation and the annealing process on the distribution of Σ3 twin boundaries in 690 alloys and found that the length fraction of the Σ3 twin boundaries increases firstly and then decreases with increasing cold strain. Tan et al. [35] reported that the grain boundary character distribution of 617 alloys can be significantly modified through TMP, and the length fraction of Σ3^n^ twin boundaries increases from this by about five times. Several such works reveal that TMP can improve the density of Σ3^n^ twin boundaries in nickel-based alloys. However, the magnitude of the deformation in the traditional TMP processing route is limited to low or medium levels, and higher twin densities rely on multiple iterations of cold deformations followed by annealing. For the Ni80A superalloy, components fabricated by the thermal deformation processes, such as electric-upsetting and forging, it is impracticable to improve the mixed grain boundary network through the existing TMP processing route.

Combining the TMP processing route for GBE is a new attempt to improve the mixed grain boundary network through a two-stage deformation method, i.e., prior cold deformation followed by thermal deformation. Further, the influencing mechanisms of prior cold deformation on the mixed grain boundary network in thermal deformation were studied systematically. Firstly, the two-stage deformation experiments for the Ni80A superalloys were conducted, wherein the specimens were subjected to cold deformation under different strains, and this was followed by isothermal compression under the same condition. After this, the flow behaviors were analyzed from the obtained true stress–strain curves, and the microstructures during the two-stage deformation process were characterized. The variations of DRX volume fraction, grain size, and twin density with prior cold strain were quantitatively analyzed. Finally, the influencing mechanisms for improving the mixed grain boundary network via a two-stage deformation method were uncovered. This investigation could provide a theoretical foundation for the design of GBE routes for high-temperature alloys.

## 2. Material and Experimental Procedures

**Figure 1:**
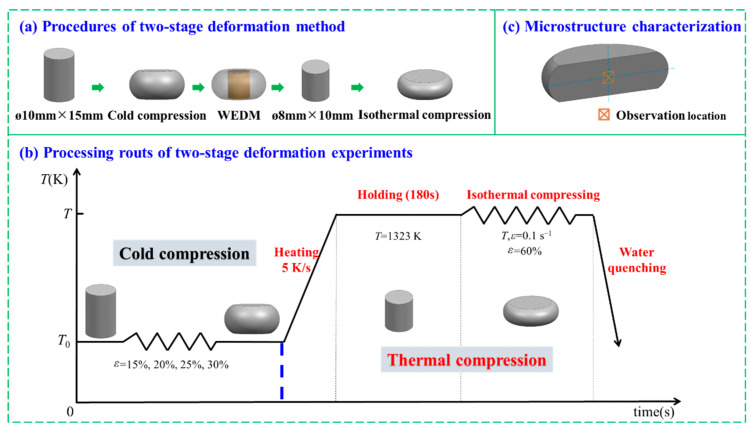
Schematic illustration of the two-stage deformation method and its processing routs.

In this work, the studied material was Ni80A superalloy, for which the detailed chemical composition (wt.%) is listed in Table 1 [22]. In this work, in order to improve the mixed grain boundary network in the thermal deformation processes of nickel-based superalloys, the two-stage deformation method was adopted. This can be divided into two stages: a prior cold deformation stage to form high-density dislocation and a subsequent thermal deformation stage to promote the occurrence of DRX processes and the formation of twins. Here, the two-stage deformation experiments for the Ni80A superalloy were conducted, and the detailed processing routes are illustrated in Figure 1. A total of nine standard cylindrical specimens, with a size of *ϕ* 10 mm × 15 mm, were cut from an as-forged billet by wire electrical discharge machining (WEDM). Four of these were prepared for two-stage deformation experiments, in which the specimens were subjected to cold deformation with different true strains, followed by thermal deformation. During the cold deformation stage, the specimens were cold compressed under the true strains of 0.163, 0.223, 0.288, and 0.357 (height reductions of 15, 20, 25, and 30%), respectively. Following this, the cold deformed specimens were machined by WEDM to obtain the standard cylindrical specimens, with a size of *ϕ* 8 mm × 10 mm. During the WEDM process, the central axis of the separated specimens should coincide with that of the cold deformed specimens. Subsequently, the separated specimens were prepared for isothermal compression experiments. During the thermal deformation stage, the separated specimens were heated to 1323 K under a heating rate of 5 K/s and held at this temperature for 180 s to ensure uniform temperature distribution. Then, the heated specimens were isothermally compressed into the fixed true strain of 0.916 (height reduction of 60%) under a strain rate of 0.1 s^−1^, immediately followed by water quenching to retain the elevated temperature microstructures. These compression experiments were carried out on a Gleeble-3500 thermo-mechanical simulator, and more experimental details are included in [22]. To characterize the microstructure evolution in cold deformation, the other four specimens were cold compressed to the height reductions of 15, 20, 25, and 30%, respectively. As a control, the remaining one specimen was only isothermally compressed at the temperatures of 1323 K under a strain rate of 0.1 s^−1^, with a true strain of 0.916 to characterize the microstructures in single thermal deformation.

Afterwards, all of the deformed specimens were symmetrically sectioned into two semi-cylinders, along the compression axis, and the geometric centers of the sectioned surfaces (as shown in Figure 1c) were used for microstructure characterization. The sectioned surfaces were ground firstly and then electro-polished in a solution of HClO_4_:CH_3_COOH:H_2_O = 1:1:8 (vol.) with a voltage of 20 V at −15 °C for 22 s. The deformed microstructures were characterized using electron back-scattered diffraction (EBSD) technology. For the specimens subjected to the two-stage deformation process, an area of 480 × 360 pixels was scanned using a step size of 0.5 μm. As for the cold-deformed specimens, an area of 520 × 390 pixels was scanned with a step size of 2 μm. Oxford instruments channel 5 software was employed to construct the EBSD maps: inverse pole figure (IPF), EBSD map highlighting the DRX volume fraction and grain boundary network, band contrast map with Σ3^n^ twin boundaries, and kernel average misorientation (KAM) map. The grain size was detected as a region being completely bounded by high angle grain boundary (HAGBs) without considering Σ3^n^ twin boundaries. The critical misorientation angles of 3° and 15° were used to identify the sub-grain and grain boundaries, respectively. The local intragranular misorientation was calculated up to the fifth nearest neighbor, with a maximum misorientation angle of 5°. The Σ3^n^ (n = 1, 2, 3) twin boundaries were identified according to the Brandon criterion [36]. The density of the Σ3^n^ twin boundaries (BLD_Σ3_^n^, μm^−1^) was introduced to evaluate the proportion of the annealing twins; this can be calculated using the equation (*N*_p_ × Δ)/*A* [37], where *N*_p_ constitutes the number of pixels of Σ3^n^ twin boundaries, Δ represents the step size in μm, and *A* represents the scanning area in μm^2^.

**Figure 2:**
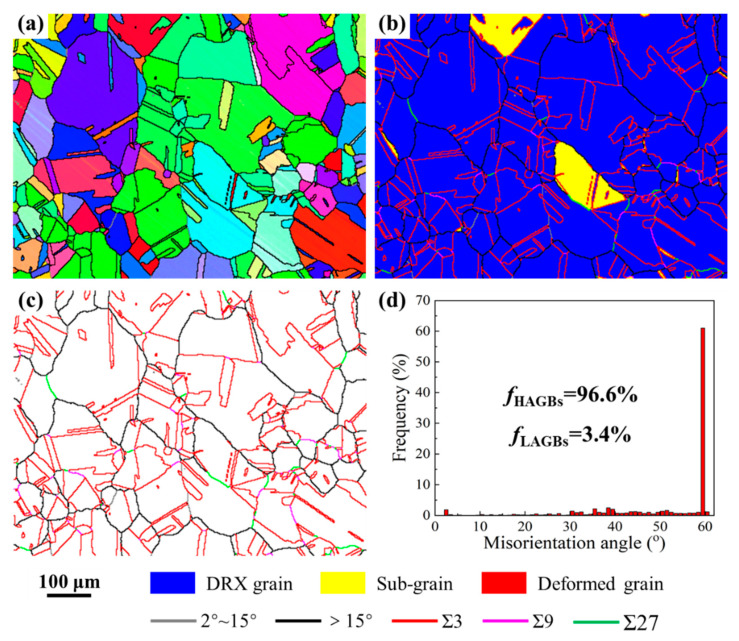
Initial microstructures of the as-received Ni80A superalloy. (**a**) IPF map; (**b**) EBSD maps highlighting DRX volume fraction and grain boundary network; (**c**) band contrast maps with Σ3^n^ twin boundaries; (**d**) distribution of misorientation angle.

Figure 2 exhibits the initial microstructures of the as-received Ni80A superalloy. It shows that the grain orientation is basically even (Figure 2a). The initial microstructures consist of equiaxed DRX grains with an average size of 34.8 µm, and the DRX volume fraction accounts for 94.9% (Figure 2b). In addition, the random HAGBs are adequately interrupted by the Σ3^n^ twin boundaries (Figure 2c). The proportion of HAGBs with misorientation angles greater than 15° accounts for 96.6% of the whole of the grain boundaries, while the proportion of low angle grain boundaries (LAGBs) with misorientation angles of 2~15° is 3.4% (Figure 2d).

## 3. Results and Discussion

### 3.1. Characterization of the Flow Behaviors from the True Stress–Strain Curves

**Figure 3:**
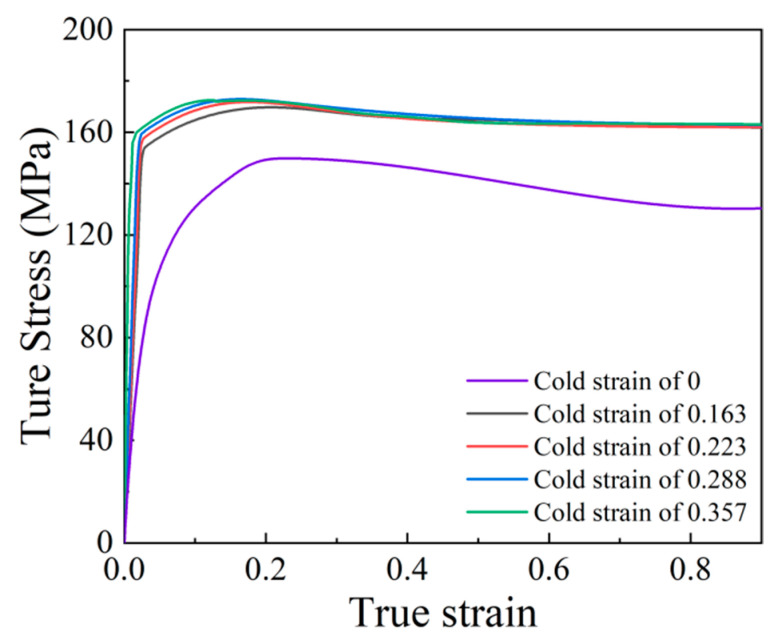
True stress–strain curves of the specimens with different cold strains subjected to thermal deformation at the temperature of 1323 K and a strain rate of 0.1 s^−1^.

Figure 3 displays the true stress–strain curves of the Ni80A superalloy derived from the isothermal compression experiments, in which the specimens with different cold strains were isothermally compressed at the temperature of 1323 K and a strain rate of 0.1 s^−1^. It was found that all of these true stress–strain curves exhibit the typical characteristics of DRX-type flow behaviors, which can be divided into three stages, including the work hardening (WH)-stage, softening stage, and the steady stage. In the first stage, since dynamic recovery (DRV) is too weak to offset the WH induced by the multiplication and accumulation of the dislocations, flow stress increases sharply with strain increasing. In the second stage, when the accumulated dislocation density exceeds a critical value, i.e., the strain reaches the critical strain for the onset of DRX, DRX takes place and then accelerates the annihilation of the dislocations. Under the competition behavior of WH and DRV as well as DRX, the flow stress continuously increases to a peak; meanwhile, its increasing rate decreases. When the flow stress reaches peak stress, the softening behaviors caused by DRX and DRV are enough to overcome WH. With the continuously increasing strain, DRX takes the predominant role in the softening behaviors, thus, resulting in the decreasing of flow stress. Finally, the flow stress keeps a steady state due to the dynamic equilibrium between WH and softening. In addition, from Figure 3, it is also seen that the flow behaviors are significantly influenced by prior cold deformation. By comparing the curves with one another, it can be easily found that with the increase of cold strain, flow stress increases and the corresponding WH stage is shorter.

**Figure 4:**
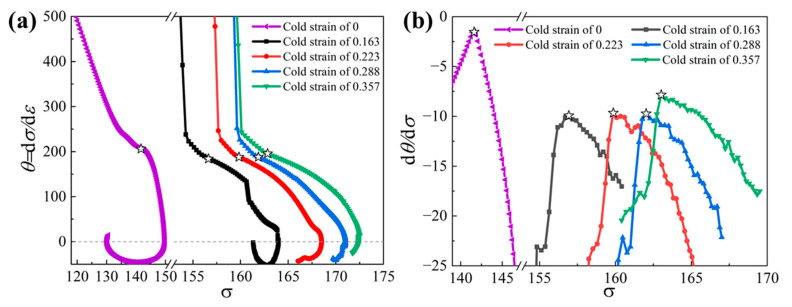
Curves of *θ* − *σ* (**a**) and d*θ*/d*σ* versus *σ* (**b**) of the specimens with different cold strains, subjected to thermal deformation at the temperature of 1323 K and a strain rate of 0.1 s^−^^1^.

From the obtained true stress–strain curves in Figure 3, two characteristic points indicating the existence of apparent DRX behaviors, i.e., the critical strain for the onset of DRX and the strain for peak stress, were introduced to analyze the impact of prior cold strain on DRX behaviors. By taking the derivative of each true-stress curve with respect to strain, the work hardening rate, θ=dσ/dε, can be calculated. The variations of work hardening rate, θ, with stress, σ, are plotted in Figure 4a. Then, by taking the derivative of each, θ−σ, curve, with respect to stress, the variations of d*θ*/d*σ* with σ were plotted (Figure 4b). According to Figure 4, when the value of d*θ*/d*σ* reaches the maximum, which corresponds to the inflection point of *θ* − *σ* curves, the critical stress for DRX initiation can be identified, and then the critical strain, $\varepsilon_{c}$, can be obtained. The peak strain, *ε*_p_, can be identified from the true stress–strain curves directly. The obtained critical strain, $\varepsilon_{c}$, and peak strain, *ε*_p_, are listed in Table 2. From Table 2, it is shown that, when the specimen (without prior cold deformation) is deformed at the temperature of 1323 K and a strain rate of 0.1 s^−1^, the critical strain and peak strain are identified as 0.080 and 0.230, while the values of these two points decreases significantly when the specimens are subjected to two-stage deformation. This indicates that DRX is activated in advance under the effect of prior cold deformation, while the effect gets stronger with increasing cold strain. In addition, it can be inferred from Table 2 that the microstructure with finer grain size and higher DRX degree will be obtained after a two-stage deformation in comparison to the microstructures formed after a single thermal deformation. In the following sections, the microstructure evolution during the two-stage deformation process of the Ni80A superalloy will be characterized and discussed.

### 3.2. Evolution of Microstructures in Cold Deformation

**Figure 5:**
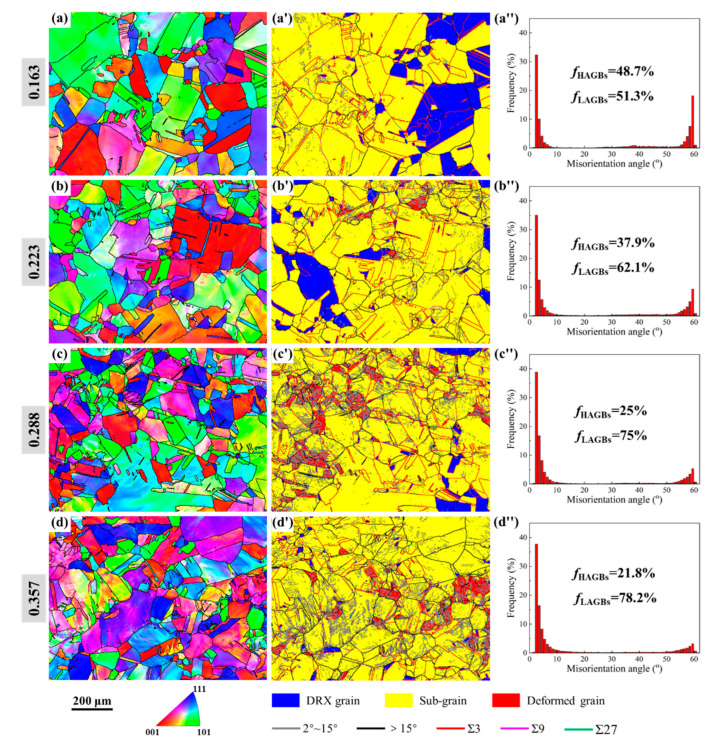
Microstructure characterization results after cold deformation with different strains: 0.163, 0.223, 0.288, and 0.357. (**a**–**e**) IPF maps; (**a**′–**e**′) EBSD maps, highlighting the DRX volume fraction and grain boundary network; (**a**″–**e**″) distribution of misorientation angle.

Figure 5 illustrates the microstructures of the specimens that were cold compressed to the true strains of 0.163, 0.223, 0.288, and 0.357 (height reductions of 15, 20, 25, and 30%, respectively). From the IPF maps in Figure 5a–d, it can be seen that the grain orientation has changed, which shows obvious plastic deformation characteristics. With an increase in cold deformation, the equiaxed grains in the initial microstructures are gradually elongated, and the grain orientation becomes more and more uneven. Figure 5a′–d′ represent the obtained EBSD maps, highlighting the DRX volume fraction and grain boundary network. It is clearly shown that the DRX grains in the initial microstructures gradually transform into sub-grains. When the specimens were cold compressed to a strain of 0.223, 0.288, and 0.357, the deformed grains increase sharply, and more broken LAGBs appear in the sub-grains and deformed grains, resulting in a significant difference in grain orientation (Figure 5b–d). From the misorientation angle distributions in Figure 5a″–d″, it is noted that when the strain increases from 0.105 to 0.357, the proportions of HAGBs are measured as 48.7, 37.9, 25, and 21.8%, while the proportions of the LAGBs correspond to 51.3, 62.1, 75, and 78.2%, respectively. This indicates that with cold strain increasing, the proportion of HAGBs decreases gradually, while the proportion of LAGBs shows an opposite trend.

**Figure 6:**
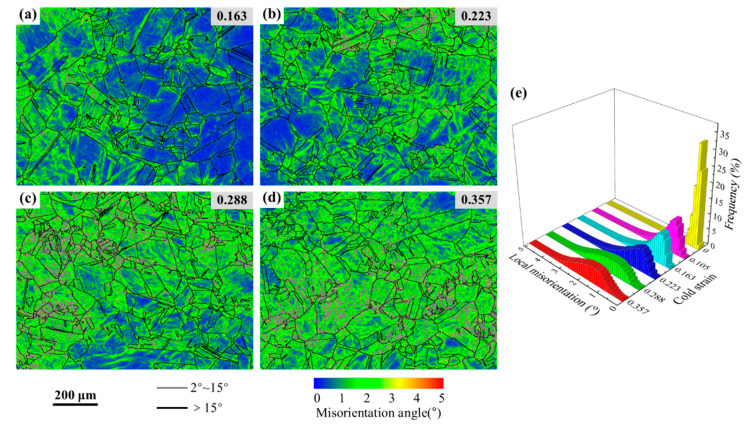
KAM maps (**a**–**d**) and local intragranular misorientation distributions (**e**) of the specimens cold compressed to different strains of 0.163, 0.223, 0.288, and 0.357.

A kernel average misorientation (KAM) map is usually used to qualitatively reflect the uniformity of plastic deformation. The microstructures with higher KAM values indicate a greater degree of plastic deformation or higher dislocation density [38]. Figure 6a–d exhibit the KAM maps of those specimens cold compressed to different strains. It was found that the local intragranular misorientation inside of the grains changes obviously, after cold deformation. When deformed at higher cold strain, there exists an enormous difference in local intragranular misorientation in the microstructures. In order to intuitively analyze the local intragranular misorientation characteristics, the corresponding boundary length fractions of the local intragranular misorientations were statistically computed and plotted as a 3-D histogram, as shown in Figure 6e. In Figure 6e, it is evident that the local intragranular misorientation within the initial microstructures is concentrated at 0~1°, and its average KAM value is 0.26°. At the cold strains of 0.163, 0.223, 0.288, and 0.357, the average KAM values were calculated as 0.93, 1.20, 1.67, and 1.73°, respectively. With cold strain increasing, dislocation density and stored energy increase gradually. More and more sub-grains and deformed grains with abundant LAGBs are formed (Figure 5), and the local intragranular misorientation inside these grains also increases.

### 3.3. Evolution of Mixed Grain Boundary Network in Thermal Deformation

In this section, the microstructures after two-stage deformation were characterized; the specimens with different cold strains were subjected to thermal deformation under a temperature of 1323 K and a strain rate of 0.1 s^−1^ with the fixed true strain of 0.916. The influence of prior cold deformation on the mixed grain boundary network in thermal deformation was discussed. This contributes toward uncovering the mechanisms for improving mixed grain boundary network in the thermal deformation of Ni80A superalloy.

#### 3.3.1. Development of Sub-Grain Network

**Figure 7:**
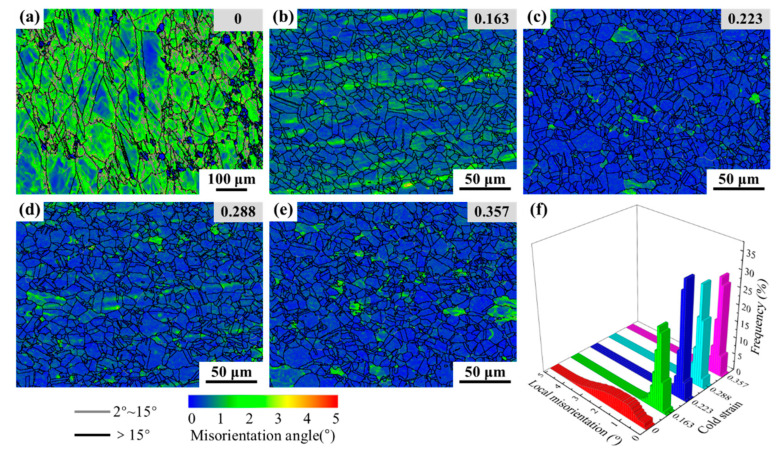
KAM maps (**a**–**e**) and local intragranular misorientation distributions (**f**) after two-stage deformation with different prior cold strains and the same thermal deformation condition.

Aiming at the development of sub-grain network in thermal deformation, the KAM maps were characterized, as shown in Figure 7a–e. Figure 7a illustrates the microstructures of the specimen subjected to a single thermal deformation at a temperature of 1323 K and a strain rate of 0.1 s^−1^. In Figure 7a, the necklace-like microstructures can be observed. There are tiny amounts of HAGBs and an abundant amount of LAGBs. An uneven and large local intragranular misorientation appears in the sub-grains. Comparing Figure 7b–e with Figure 7a, the obvious difference is that the microstructures, after two-stage deformation, were characterized as lacking local intragranular misorientation. Figure 7f shows the length fractions of the local intragranular misorientation, corresponding to Figure 7a–e. For the different cold strains (from 0 to 0.357), the average KAM values were calculated as 1.63, 0.47, 0.42, 0.40, and 0.37°, respectively. The results indicate that, with an increase in cold strain, the local intragranular misorientation in the microstructures after a two-stage deformation become smaller and smaller. It is well accepted that the LAGBs are inherently associated with sub-grain structures and dislocations. Accompanying the occurrence of DRX in the thermal deformation process, the formed LAGBs are further developed into HAGBs or Σ3^n^ twin boundaries, resulting in a lower local intragranular misorientation in the microstructures. It also can be inferred that DRX has occurred more completely under the effect of prior cold deformation.

#### 3.3.2. DRX Volume Fraction and Grain Size Evolution

**Figure 8:**
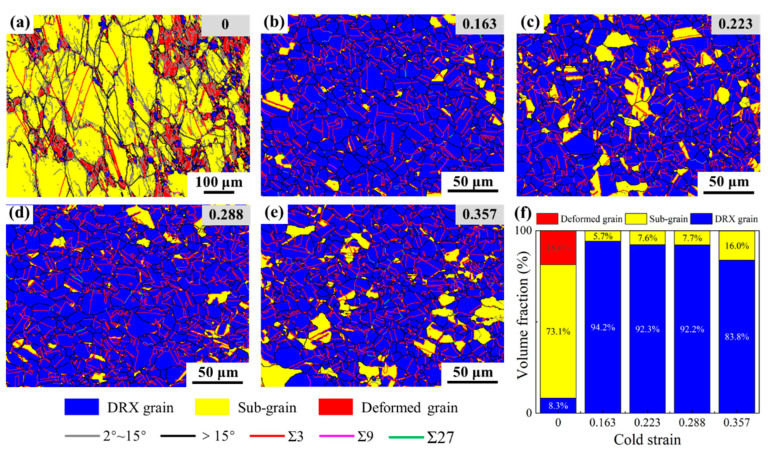
EBSD maps, highlighting the DRX volume fraction and grain boundary network (**a**–**e**) and the variations of the volume fractions of the DRX grain, sub-grain, and deformed grain, (**f**) after two-stage deformation with different prior cold strains and the same thermal deformation condition.

Figure 8a–e illustrates the EBSD maps, highlighting the DRX volume fraction and grain boundary network after a two-stage deformation with different prior cold strains and the same thermal deformation condition. In Figure 8b–e, the microstructures after a two-stage deformation are characterized by having a large number of equiaxed DRX grains and only a few sub-grains. In comparison to the necklace-like microstructures of the specimen without cold deformation (Figure 8a), the microstructures, after two-stage deformation, are significantly modified. The volume fractions of DRX grain, sub-grain, and deformed grain, after thermal deformation, were calculated, and their variations with cold strain are demonstrated in Figure 8f.

In Figure 8f, it can be clearly seen that, for the microstructures, after a single thermal deformation, the volume fractions of the sub-grain and deformed grain account for 73.1% and 18.6%, respectively, while the DRX volume fraction accounts for only 8.3%. As for the microstructures, after two-stage deformation, the volume fractions of the sub-grain and deformed grain decrease, and the DRX volume fraction significantly increases and exceeds more than 80%. This confirms that DRX occurs more completely under the effect of prior cold deformation. Combining the microstructure characterization results for cold deformation and the analysis of flow behaviors for thermal deformation, the reasons for the improved DRX volume fraction after a two-stage deformation can be explained as follows. As is known to all, DRX is a thermal-activation process which requires a certain incubation period before the DRX process can occur. Under a temperature of 1323 K and a strain rate of 0.1 s^−1^, when the specimen is subjected to a single thermal deformation, the grain boundary migration rate is relatively slow, and there is not enough stored energy to fully activate the DRX process, resulting in the longer incubation period. However, for those specimens subjected to a two-stage deformation, it can be seen that the sub-grain boundaries with high-density dislocation form within the microstructures after cold deformation (Figure 6). In the following thermal deformation process, these formed sub-grain boundaries with higher stored energy will become the favorable nucleation sites of the DRX grains and twins, thereby accelerating their nucleation. At the same time, the driving force of grain boundary migration increases under the effect of prior stored energy, with DRX being activated in advance; this contributes to the occurrence of the DRX process and the annihilation of dislocation. Consequently, the DRX volume fraction after two-stage deformation increases.

Nevertheless, it is noticeable that when the cold strain exceeds 0.288, with an increase of cold strain, the sub-grain volume fraction increases while the DRX volume fraction decreases. Although more and more sub-grain boundaries with high-density dislocation are formed in cold deformation, there is no sufficient thermal activation energy to promote all the formed sub-grains to transform into DRX grains. To sum up, it can be concluded that, under the same thermal deformation condition, with the increase of cold strain, the DRX volume fraction increases firstly and then gradually reduces, while the sub-grain volume fraction demonstrates an opposite trend.

**Figure 9:**
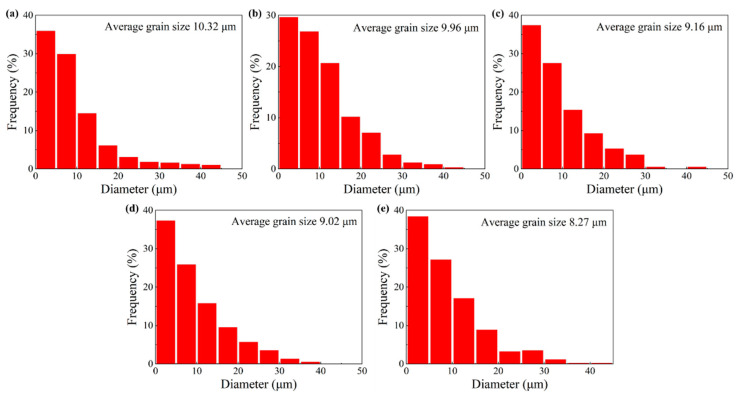
Grain size distributions after two-stage deformation with different prior cold strains and the same thermal deformation condition. (**a**) 0; (**b**) 0.163; (**c**) 0.223; (**d**) 0.288; (**e**) 0.357.

Excluding the Σ3^n^ twin boundaries, grain size distributions, after two-stage deformation, were also acquired, as depicted in Figure 9a–e. It is clearly found that the grain size distributions, after two-stage deformation, hugely contrast with the distribution without prior cold deformation. In Figure 9a, although the area fraction of the finer grains with a size lower than 10 µm accounts for 30%, the grain size distribution is uneven. When the cold strain increases from 0.163 to 0.357, more uniform grain size distributions, after two-stage deformation, can be seen in Figure 9b–e. At the cold strains of 0.163, 0.223, 0.288, and 0.357, the area fraction of finer grains with a size lower than 10 µm are 56.6, 65, 63.2, and 65.6%, respectively. The average grain size values without considering the Σ3^n^ twin boundaries are measured as 9.96, 9.16, 9.02, and 8.27 µm, respectively. This reveals that an increase in cold strain can obviously increase the area fraction of the finer grains and decrease the average grain size. When combining the microstructure characterization results from Figure 6, the reasons for this trend can be explained. As cold deformation increases, the dislocation density and prior stored energy increase. Under the effect of prior stored energy, the DRX process is activated in advance and occurs more completely, resulting in grain refinement after two-stage deformation. In addition, it should be noted that when the cold strain exceeds 0.288, although the DRX volume fraction decreases, the grain size is refined continuously. It is deduced from this that, at a higher cold strain, the nucleation sites for the DRX grains increase. More and more new sub-grains are generated and tend to transform into DRX grains, while the space for the growth and expansion of the DRX grains is restricted (by each other). Consequently, the average grain size decreases with an increase in cold strain.

#### 3.3.3. Twin Boundary Characteristics and Its Density Evolution

**Figure 10:**
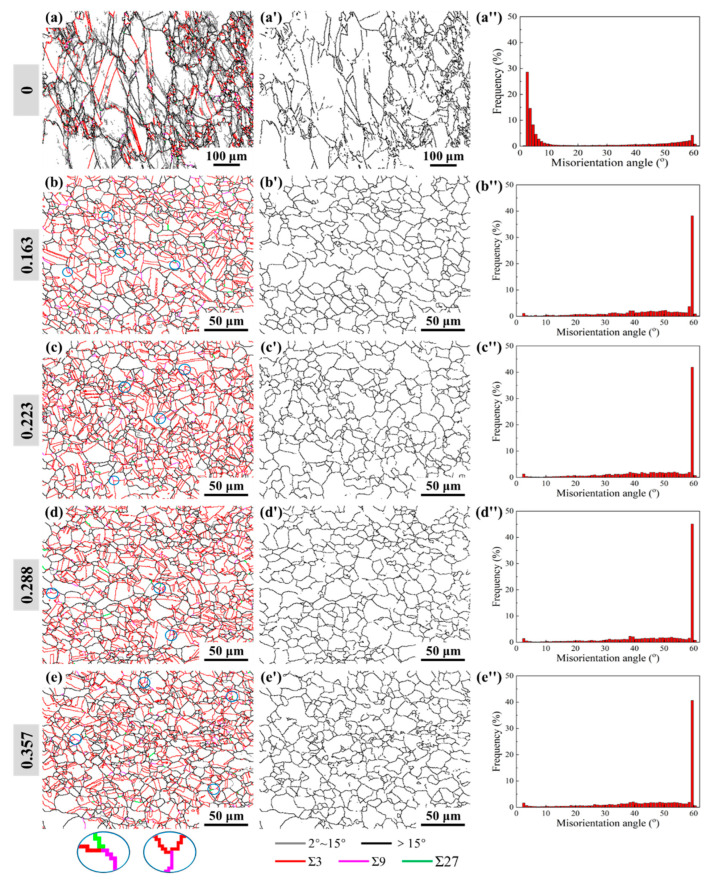
Microstructure characterization results after two-stage deformation with different prior cold strains and the same thermal deformation condition. (**a**–**e**) Band contrast maps with Σ3^n^ twin boundaries; (**a**′–**e**′) random HAGBs connectivity maps; (**a**″–**e**″) distribution of misorientation angle.

To intuitively analyze the characteristics of the Σ3^n^ twin boundaries in the microstructures, after two-stage deformation, the band contrast maps with the Σ3^n^ twin boundaries were reconstructed, as shown in Figure 10a–e. In addition, the corresponding random HAGB connectivity maps are also illustrated in Figure 10a′–e′. As observed from Figure 10b′–e′, for the microstructures, after two-stage deformation, the random HAGB networks are adequately interrupted by the Σ3^n^ twin boundaries, forming a large number of grain clusters. Compared with the microstructures from a single thermal deformation process (Figure 10a), the proportion of Σ3^n^ twin boundaries increases sharply. Moreover, the proportions of Σ9 and Σ27 twin boundaries are also relatively high, and the phenomenon of multiple twinning is obvious (Figure 10b–e). Combing Figure 10b–e with Figure 8b–e, an obvious feature is that the new DRX grains are formed and accompanied by abundant twins, presenting highly twinned-grain structures. Besides, the Σ3^n^ twin boundaries are mostly distributed in the newly generated DRX grains instead of the sub-grains. These results imply that the formation of twins is strongly associated with DRX behavior during two-stage deformation, and the probability of forming twins increases with the occurrence of the DRX process. Figure 10a″–e″ exhibit the corresponding distributions of the misorientation angle. It is apparent that the proportion of LAGBs in Figure 10a” is relatively high, while the proportion of HAGBs is dominant in Figure 10b″–e″. The most obvious reason for this phenomenon is that the occurrence of DRX and the formation of twins contribute to promoting LAGBs turning into HAGBs or Σ3^n^ twin boundaries. Thence, the microstructures, after two-stage deformation, are dominated by HAGBs. While abundant LAGBs are characterized within the single thermal deformation microstructures as a result of the incomplete occurrence of the DRX process and the lower probability of forming twins.

**Figure 11:**
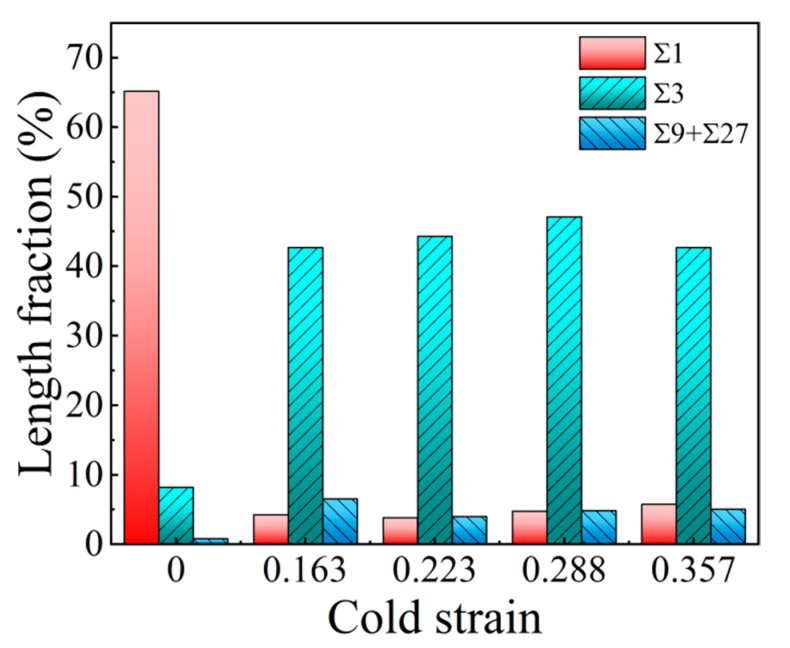
Variations of length fractions of Σ1 grain boundaries and Σ3^n^ twin boundaries with cold strain.

In order to quantitatively analyze the evolution of the Σ3^n^ twin boundaries after two-stage deformation, the length fractions of the Σ1 (LAGBs), Σ3, Σ9, and Σ27 twin boundaries were statistically analyzed, with their variations against cold strain shown in Figure 11. From Figure 11, when the specimens are deformed by the proposed two-stage deformation method, the length fraction of the Σ1 grain boundaries decreases significantly, while the length fractions of the Σ3 and Σ9 + Σ27 twin boundaries increase remarkably. This is mainly because DRX happens in advance and more completely under the effect of prior cold deformation. Correspondingly, the probability of accident growth will increase, thus, provoking the formation of Σ3 twin boundaries. As indicated by the circles in Figure 10b–e, with a higher proportion of Σ3 twin boundaries, multiple twinning, such as Σ9 and Σ27 twin boundaries, forms by following rules concerning the joining or dissociation of the Σ3^n^ twin boundaries: Σ3 + Σ3 = Σ9, Σ3 + Σ9 = Σ3, Σ3 + Σ9 = Σ27 [39,40,41]. As a result, the length fraction of the Σ9 + Σ27 twin boundaries is correspondingly high after two-stage deformation. In addition, it is worth noting that, as cold deformation increases, the length fraction of the Σ3 twin boundaries increases firstly and then decreases gradually, reaching a maximum of 47.1% at a cold strain of 0.288. When combining Figure 11 with Figure 8f, it is found that the DRX volume fraction decreases when the cold strain exceeds 0.288, and the probability of accidental growth during DRX process decreases as well.

**Figure 12:**
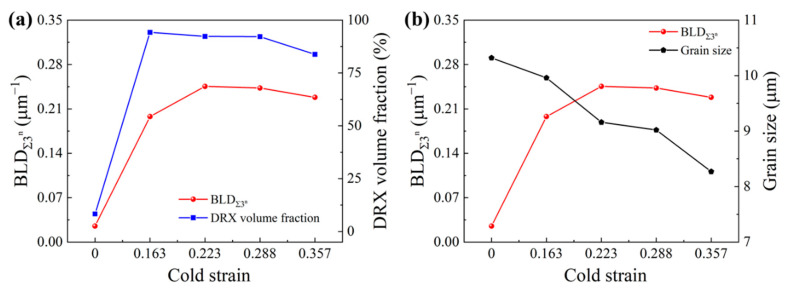
Relationships of BLD_Σ3_^n^ with DRX volume fraction (**a**) and grain size (**b**) varying with cold strain.

Additionally, the density of the Σ3^n^ twin boundaries (BLD_Σ3_^n^), after two-stage deformation, was estimated. The relationships of BLD_Σ3_^n^ with the DRX volume fraction and grain size were also demonstrated, as in Figure 12a,b, respectively. From Figure 12, when the cold strain increases from 0 to 0.357, the values of BLD_Σ3_^n^ were calculated as 0.0252, 0.1980, 0.2455, 0.2429, and 0.2282 μm^−1^, respectively. Compared to the BLD_Σ3_^n^ value, after a single thermal deformation, the BLD_Σ3_^n^ values after two-stage deformation increase remarkably, improving by about nine times, with the cold strain of 0.223. It can also be noted from Figure 12 that BLD_Σ3_^n^ evolution is synthetically affected by the DRX volume fraction and grain size. In Figure 12a, the variation of BLD_Σ3_^n^ exhibits an approximately similar tendency to that of the DRX volume fraction. In Figure 12b, there is non-liner relation between BLD_Σ3_^n^ and grain size; with an increase in cold strain, grain size decreases constantly, while BLD_Σ3_^n^ increases firstly and then decreases gradually. On the one hand, during grain boundary migration, the probability of forming Σ3 twin boundaries increases with the occurrence of DRX. On the other hand, the formation of Σ3 twin boundaries can accelerate the bulging of the original grain boundary, leading to the newly formed DRX grains separating from the parent grains, contributing to the nucleation and development of the DRX grains [14,17,18]. However, when the cold strain exceeds 0.288, the DRX volume fraction decreases; meanwhile, the space for the growth and expansion of the DRX grains is restricted, decreasing the probability of Σ3^n^ twin boundaries being formed. This is why BLD_Σ3_^n^ decreases with decreasing grain size.

Based on the above experimental results and analysis, it can be concluded that the mixed grain boundary network in the thermal deformation of Ni80A superalloy can be significantly modified through a two-stage deformation method. The microstructures with higher BLD_Σ3_^n^ and finer grain size in the thermal deformation process can be obtained by adjusting the prior cold deformation.

### 3.4. Mechanism for Improving Mixed Grain Boundary Network

**Figure 13:**
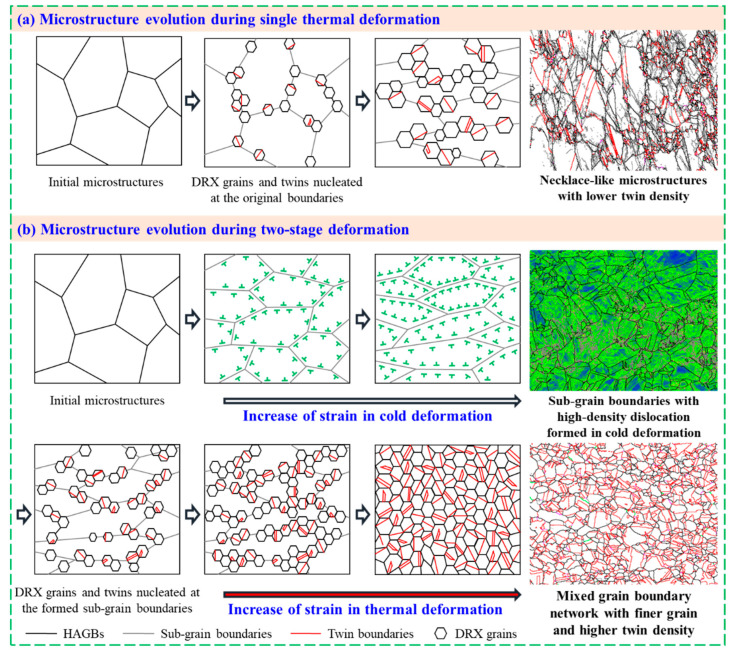
Schematic representation of microstructure evolution during single thermal deformation and two-stage deformation.

During the dynamic thermal deformation process of nickel-based alloys, DRX behaviors and twin evolution mechanisms are strongly associated with stored energy, influenced by the processing parameters, including temperature, strain, and strain rate [20,22,32,42]. It is well accepted that DRX grains usually nucleate at the bulging of the original grain boundaries. The stored energy in the grain boundaries comes from the dislocation density difference between the sub-grains and grains, and it grows rapidly to a DRX activation energy as deformation increases. To clearly describe the influencing mechanisms of prior cold deformation on the mixed grain boundary network in the thermal deformation process of Ni80A superalloy, a schematic representation of microstructure evolution during a single thermal deformation and a two-stage deformation process is shown in Figure 13. As depicted in Figure 13a, when the specimen is subjected to single thermal deformation at a relatively low temperature and an intermediate strain rate of 0.1 s^−1^, the necklace-like microstructures, with lower twin density, are formed. This is mainly due to the fact that the stored energy for activating the DRX process is insufficient; meanwhile, the probability of forming twins decreases under the relatively slow grain boundary migration rate [22,29]. When the specimens are subjected to two-stage deformation, the microstructures improve and are characterized as the mixed grain boundary network, with a finer grain and higher twin density, as illustrated in Figure 13b. In the cold deformation stage, the sub-grain boundaries, with high-density dislocation, are formed. In the subsequent thermal deformation stage, the formed sub-grain boundaries can provide favorable nucleation sites for DRX grains and twins. Meanwhile, the driving force for grain boundary migration is enhanced due to the prior stored energy from cold deformation. In these cases, the critical strain for DRX initiation and the peak strain is lower than that within single thermal deformation (Figure 4). Correspondingly, DRX occurs more prematurely and completely, thus, promoting the formation of twins. With that, the mixed grain boundary network, with finer grain and higher twin density, are obtained.

Based on the analysis above, it can be confirmed that, for a fixed thermal deformation condition, the mixed grain boundary network with a finer grain and higher twin density can be achieved by adjusting the prior cold deformation. It is worth mentioning that the thermal deformation temperature and strain rate will also affect the grain boundary migration in the thermal deformation process; the mixed grain boundary network in thermal deformation may be improved by comprehensively coordinating the thermal deformation parameters and the prior cold deformation. Further study and discussion of the effect of the thermal deformation parameters will be carried out in future work. Following this, the quantitative models describing the evolution of grain size and twin density in the two-stage deformation process will be developed to precisely adjust the cold and thermal deformation parameters for achieving the desired microstructures of the alloys. In addition, the cold deformation experiments in the present work were performed at room temperature. It is worth studying whether the lower cold deformation temperature can improve the mixed grain boundary network in the thermal deformation processing of alloys more significantly.

## 4. Conclusions

In this work, a two-stage deformation method, i.e., prior cold deformation followed by thermal deformation, was proposed to achieve a mixed grain boundary network with a higher density of Σ3^n^ twin boundaries in the thermal deformation process of alloys. The influencing mechanisms of the prior cold deformation on the mixed grain boundary network from the thermal deformation of Ni80A superalloy were investigated. Some conclusions drawn from this study are as follows.

(1) The flow behaviors of Ni80A superalloy are significantly influenced by prior cold deformation. The critical strain for the onset of DRX and peak strain decreases remarkably under the effect of prior cold deformation, implying that DRX is activated in advance. These two indicators decrease with increasing cold strain;
(2) In comparison to the necklace-like microstructures from the single thermal deformation, the microstructures of the two-stage deformation process are characterized by finer and equiaxed DRX grains accompanied by abundant Σ3^n^ twin boundaries and a significantly improved value of BLD_Σ3_^n^ of about nine times. With increasing cold strain, the grain size decreases continuously, while BLD_Σ3_^n^ increases at first and then decreases gradually;
(3) The variations of BLD_Σ3_^n^ against prior cold strain exhibit an approximately similar tendency to that of the DRX volume fraction, with it increasing at first and then decreasing gradually with grain refinement. This indicates that the relationship between BLD_Σ3_^n^ and grain size is non-liner. The mixed grain boundary network, with higher BLD_Σ3_^n^ and finer grains under thermal deformation, can be obtained by adjusting the prior cold deformation;
(4) The mechanisms for improving the mixed grain boundary network by a two-stage deformation method have been uncovered. The sub-grain boundaries formed in prior cold deformation stimulate the nucleation of the DRX grains and twins; meanwhile, the driving force for grain boundary migration is enhanced due to the prior stored energy. In these cases, DRX is activated in advance and occurs more completely, thereby promoting the formation of Σ3^n^ twin boundaries.

## Figures and Tables

**Table 1:** Chemical composition of the studied Ni80A superalloy (wt. %).

Element	Cr	Fe	Ti	Mn	Si	Al	C	Ni
Content	20.87	1.26	2.07	0.63	0.55	0.68	0.069	Balance

**Table 2:** Values of critical strain, $\varepsilon_{c}$, and peak strain, $\varepsilon_{p}$, under different cold strains.

Cold Strain	$\mathrm{Critical}\;\mathrm{Strain}\;\varepsilon_{c}$	$\mathrm{Peak}\;\mathrm{Strain}\;\varepsilon_{p}$
0	0.080	0.230
0.163	0.042	0.209
0.223	0.038	0.177
0.288	0.037	0.166
0.357	0.033	0.127

## Data Availability

Not applicable.
